# Machine learning-based adaptive personalization in virtual reality stroke rehabilitation: a systematic review

**DOI:** 10.3389/fresc.2026.1827658

**Published:** 2026-06-16

**Authors:** Arar Al Tawil, Siti Hazyanti Mohd Hashim, Aseel Aburub, Mohammad Z. Darabseh, Viktória Prémusz, Márta Hock

**Affiliations:** 1 School of Computer Sciences, Universiti Sains Malaysia, USM Penang, Malaysia; 2 Department of Computer Science, Faculty of Information Technology, Applied Science Private University, Amman, Jordan; 3 Department of Physiotherapy, Faculty of Allied Medical Sciences, Applied Science Private University, Amman, Jordan; 4 Department of Physiotherapy, School of Rehabilitation Sciences, University of Jordan, Amman, Jordan; 5 Physical Activity Research Group, János Szentágothai Research Center, University of Pécs, Pécs, Hungary; 6 Institute of Physiotherapy and Sports Science, Faculty of Health Sciences, University of Pécs, Pécs, Hungary

**Keywords:** adaptive personalization, machine learning, reinforcement learning, stroke rehabilitation, virtual reality

## Abstract

**Background:**

Stroke is the second leading cause of disability worldwide. Recently, VR has been seen as a new therapeutic tool. Many existing VR systems rely on rule-based difficulty adjustment that may not adequately capture the non-linear dynamics of stroke recovery. Machine learning algorithms can provide adaptive personalization in virtual reality stroke rehabilitation.

**Objective:**

The aim is to systematically review available evidence on ML-based adaptive personalization mechanisms in VR stroke rehabilitation, focusing on algorithms and adaptation strategies, clinical outcomes and implementation considerations.

**Methods:**

In accordance with the PRISMA 2020 statement six databases were searched (January 2015-December 2025). Included studies conducted with the use of ML algorithms for VR. The ML algorithms, adaptation mechanisms, therapeutic parameters, clinical outcomes, and implementation factors are covered in data extraction. Risk of bias was assessed with validated tools; meta-analysis was performed when appropriate.

**Results:**

In total, twenty-five studies were included for analysis. Reinforcement learning (*n* = 8) and deep learning (*n* = 7) were prominent algorithms. Around 80% of studies implemented real-time closed-loop adaptation. The results of the meta-analysis show that the upper limb motor function (FMA-UE) improved by 7.47 points compared with the control group (MD: 7.47 points; 95% CI: 5.38–9.57; *p* < 0.001). This improvement exceeds the minimal clinically important difference. The usability scores were positive (SUS=72.5), with few adverse events reported.

**Conclusion:**

ML-based adaptive VR rehabilitation demonstrates clinical efficacy and safety for stroke recovery, though implementation barriers in resource-limited settings require attention.

**Systematic Review Registration:**

https://www.crd.york.ac.uk/PROSPERO/view/CRD420261298450, PROSPERO CRD420261298450.

## 1 Introduction

### 1.1 Background and rationale

One of the leading causes of long-term disability globally is stroke, which occurs in 13.7 million people a year. Moreover, stroke causes a significant, persistent motor, cognitive and functional disability. Stroke contributes to the worsening of the quality of life (1). The Global Burden of Disease Study has shown that stroke is the third leading cause of death and the second leading cause of disability in individuals over the age of 50 years (2). According to author data (3), 55%–75% of stroke survivors experience motor deficits after the event with a control deficit, fine motor deficit or dual-task performance ability deficit. The remaining deficits will later burden the health care system, caregivers and patients themselves, thus highlighting the need for effective rehabilitation strategies to optimize the functional recovery path.

According to Dimyan and Cohen (4), plasticity is the ability of the central nervous system to “alter its structure and function dynamically, responding to experience, and to sensory feedback”. Stroke recovery involves cortical reorganization and compensatory network recruitment rather than true neural regeneration. The strength of synaptic connections and the axonal rearrangement of cortical pathways determine the neural mechanisms of such plasticity (4). To effectively target neural plasticity through rehabilitation, interventions must provide intensive, repetitive and goal-oriented practice that is appropriately scaled to the individual patient (5). The time course of recovery is not linear with the most significant improvements happening in the first three to six months of injury when neuroplasticity is at its peak. Thereafter, this process is slower and gains become small, depending on the intensity of training and motivation (6).

Stroke rehabilitation methods that have shown clinical validity are limited due to a lack of ecological validity, low treatment intensity, and difficulty in patient engagement over time (7). Conventional physical therapy programs are impeded by ecological parameters such as the duration and frequency of the intervention, geographic access, number of available caregivers along with subjective parameters like patient motivation and perseverance for training (8). These constraints have increased interest in new, technology-based rehabilitation methods, with virtual reality (VR) emerging as a promising intervention paradigm that overcomes many limitations of traditional rehabilitation therapies.

Virtual reality is defined as a computer-based technology that enables users to interact with multisensory simulated environments and receive real-time feedback on performance (3). Rehabilitation through virtual reality (VR) can provide intensive task-oriented practice in an environment that is safe, but also ecologically valid i.e., resembles real life (9). The technology utilizes the essential motor learning principles known to be effective for skill retention including frequency, intensity, repetition, and task-focused training (10). The VR system enables implementation of multiple neurorehabilitation principles in therapeutic protocols, while also facilitating manipulation of practice conditions to optimize learning processes and experience-dependent neuroplasticity processes (11).

Laver et al. (12) carried out the most up-to-date Cochrane systematic review, which included 190 randomized controlled trials with 7,188 participants, which represents the most comprehensive evidence synthesis to date. There is moderate-certainty evidence that VR interventions improve upper limb function when added to usual care. Furthermore, there is evidence that VR may have a small benefit over other forms of therapy on arm function and balance and activity limitation. According to Khan et al. (13), upper limb, lower limb, gait, and balance recovery is further boosted by the addition of VR to conventional therapy, according to meta-reviews. According to a recent review and meta-analysis report that specifically focused on immersive VR, significant effects has been found on the Fugl-Meyer Assessment Upper Extremity (FMA-UE), a mean difference of 3.04 points (95% CI: 1.46–4.62, *p* < 0.001) (14).

A major limitation of the current Virtual Reality rehabilitation systems is their ability to provide personalized rehabilitation. Stroke patients present with different types of impairments that require unique therapies to suit their abilities, rates of progression and goals of rehabilitation (15). The conventional rule-based mechanisms tailored for adjusting difficulty have witnessed improvements over static protocols; however, they fail to represent the complex non-linear dynamics of motor recovery and may not be able to cope with variations in performance within a session (16). The effectiveness of rehabilitation programs is severely restricted if their difficulty levels are not properly matched with patient performance. This can lead to reduced engagement, plateaued progress, and diminished therapeutic benefit (8).

Algorithms from machine learning (ML) are a powerful method to provide personalized assistance and have been suggested for that purpose. These systems can modify parameters in real-time or between sessions by learning from patient performance (17) The blending of ML with VR rehabilitation represents a shift in focus from prefixed difficulty progressions towards adaptive delivery of interventions based on patients' responses. Deep learning networks have been applied with success to the decoding of EEG signals from stroke patients for motor imagery BCI systems attaining classification accuracies adequate for closed-loop use. The paper mentioned that accuracy of 66.36% for motor imagination classification in stroke patients has been obtained using convolutional neural networks with transfer learning and has lower computational complexity compared to conventional approach (29). More sophisticated architectures that incorporate residual connections and source localization have achieved motor task classification accuracies of up to 91.03% (30).

In VR rehabilitation, reinforcement learning algorithms have proven to be particularly effective for optimizing difficulty levels in real time. Grimm et al. (21) developed a closed-loop system based on Bayesian reinforcement learning principles, which resulted in 97% expansion of virtual training workspace for patients along with an enhancement of their range of motion and motor function in real-world settings. Studies and applications of Q-learning methods have involved transforming the virtual target location according to kinematic features as per reward policy set by the therapist (8). Dynamic difficulty adjustment (DDA) with reinforcement learning allows the game environment to alter itself in real time and does not require pre-training or prior information about patient capabilities. The methods taught here help support reinforcement learning by progressively challenging the patient according to that individual's capacity for functional recovery (21).

### 1.2 Research gap and justification

Although several systematic reviews have been conducted looking into the VR rehabilitation of stroke (12–14) or the usage of AI in rehabilitation settings more generally, none of them have specifically looked into how ML algorithms are able to enable adaptive personalized VR stroke rehabilitation. This is a major gap in the literature because understanding these mechanisms would benefit the design of next-generation rehabilitation technologies, the establishment of best practices for clinical application, and the identification of priority areas for research. The rapid increase in machine learning models and adopted VR systems in rehabilitation research and clinical practice makes the justification for this review stronger.

Recent umbrella review demonstrates that there is heterogeneity in the methods and results of existing systematic reviews on VR and stroke rehabilitation and therefore, no conclusions may be drawn which limit rehabilitation professionals' clinical decision making. Given the volume of contradictory evidence, this review focuses specifically on synthesizing the ML-based adaptation component, which distinguishes more modern intelligent systems from standard VR systems. In addition, the existing literature lacks adequate characterization of implementation considerations such as usability, safety, and barriers to clinical translation, which hinders the evidence-based integration of these technologies into clinical practice (26).

### 1.3 Objectives

The primary objective of this systematic review was to comprehensively examine the current evidence regarding ML-based adaptive personalization in VR stroke rehabilitation. Specifically, this review sought to achieve the following objectives:

**Goal 1:** Which ML algorithms are employed in adaptive VR stroke rehabilitation systems, and what performance metrics (accuracy, precision, F1-score, RMSE) are reported?
**Goal 2:** How are adaptation mechanisms implemented in terms of timing (real-time vs. between-session), logic, and system architecture?
**Goal 3:** What therapeutic parameters are adapted (difficulty level, speed, range of motion, feedback type/intensity), and how do these adaptations align with established motor learning principles?
**Goal 4:** What clinical outcomes are associated with ML-adaptive VR interventions, including motor function (FMA-UE (33), ARAT (34), BBT (35), WMFT (36)), functional independence (FIM (38), Barthel Index (39)), and quality of life measures?
**Goal 5:** What implementation considerations, including usability, safety, adverse events, barriers, and facilitators, influence clinical translation of ML-adaptive VR rehabilitation systems?

## 2 Methods

### 2.1 Protocol and registration

The PRISMA 2020 guidelines served as the basis for this systematic review. Before beginning the study, the review protocol was prospectively registered in the International Prospective Register of Systematic Reviews (PROSPERO) registration number CRD420261298450. In advance, the protocol specified the research questions, eligibility criteria, search strategy, data extraction procedures, risk of bias assessment methods, and analytical strategy. Supplementary Appendix S1 documents all protocol deviations and justifications. The PRISMA checklist was checked to ensure that the report of the review was comprehensive and transparent.

### 2.2 Eligibility criteria

The study eligibility was evaluated based on the population, intervention, comparison, outcomes, and study design (PICOS) framework, as given in Table 1. This ensures inclusion and exclusion criteria were applied systematically and transparently during screening.

**Table 1:** Eligibility criteria based on the PICOS framework.

**PICOS Element**	**Eligibility Criteria**
Population	Adult stroke survivors (aged ≥18 years) in any phase of recovery (acute, subacute, or chronic), presenting with motor and/or cognitive impairments secondary to ischemic or hemorrhagic stroke, as confirmed by clinical diagnosis or neuroimaging evidence
Intervention	Virtual reality-based rehabilitation systems incorporating machine learning algorithms (supervised learning, unsupervised learning, reinforcement learning, deep learning, or hybrid approaches) that enable adaptive personalization of therapeutic parameters in real-time or between consecutive sessions
Comparison	Conventional rehabilitation therapy, non-adaptive virtual reality rehabilitation, sham intervention, or no intervention; comparator not mandatory for inclusion to capture the full breadth of available evidence
Outcomes	At least one of: (a) ML algorithm performance metrics (accuracy, precision, recall, F1-score, RMSE); (b) motor function (FMA-UE, ARAT, BBT, WMFT); (c) functional independence (FIM [38], Barthel Index [39], mRS); (d) cognitive function (MoCA, MMSE); (e) quality of life (SF-36, SIS (37), EQ-5D [40]); (f) system usability (SUS)
Study Design	Randomized controlled trials, quasi-experimental studies, controlled clinical trials, prospective cohort studies, pilot and feasibility studies, single-arm intervention studies with pre-post measurements

Studies were excluded if either one of the following criteria applies: (a) the VR system uses rule-based or manually tuned adaptation schemes only (that do not employ ML components); (b) the VR system only technical papers that relate to the development of new algorithms, which were not clinically validated and did not take place at any point in patients; (c) studies that only involve healthy users; (d) rehabilitation only studies on non-stroke neurological conditions without a stroke subgroup with distinct analysis; (e) conference abstracts, review articles editorials, commentaries, correspondence, and shorter than three participant case reports; (f) those publications that are not in English; and (g) publication that took place prior to January 2015, which would be the period prior to the advent of substantial deep learning, reinforcement learning application in rehabilitation. Studies involving patients but primarily assessing algorithm performance without clinical outcome data were included only if adaptation mechanisms were actively tested in therapeutic contexts. The decision to include technical validation studies and single-arm designs was deliberate and is justified by the early-stage nature of this research field. Restricting inclusion to RCTs alone would have excluded the majority of available evidence and would not accurately reflect the current state of the science. This approach is consistent with methodological guidance for systematic reviews of emerging health technologies (Liberati et al., 2009; Higgins et al., 2022). To address internal validity concerns arising from this broad inclusion strategy, risk of bias is reported separately by study design in Section 3.8, enabling readers to assess the contribution of each design type to the overall evidence base. The inherent limitations of including non-randomized and single-arm studies are explicitly acknowledged in the Conclusion.

### 2.3 Databases used

Using the time frame of January 2015 to December 2025, the literature search was carried out on six electronic databases which includes: PubMed/MEDLINE, IEEE Xplore, Scopus, Web of Science Core Collection, Cochrane Central Register of Controlled Trials (CENTRAL) and ACM Digital Library. The databases selected aimed to capture the medical/clinical literature as well as relevant engineering/computer science literature given the cross-disciplinary nature of this review. Grey literature was also searched in Google Scholar (first 200 results), ProQuest Dissertations and Theses and ClinicalTrials.gov. The reference list of each included study and relevant systematic review were manually screened for further electronically uncaptured eligible records. When necessary, corresponding authors of included studies were contacted for missing data or methodological clarification.

### 2.4 Search strategy

The search strategy for the review was devised in consultation with an information specialist. MESH terms keywords were used in a single search string divided into three conceptual blocks (a) stroke and other cerebrovascular incident, (b) virtual reality technology and applications, (c) machine learning, artificial intelligence and adaptive systems. Boolean operators (AND, OR) were used to combine search terms in and between concept blocks. The search strategy was first developed in PubMed and then modified for the specific syntax and controlled vocabulary of each database. Supplementary Appendix S2 comprises the full search strategies of all the databases.

#### 2.4.1 Database search strategy

**Concept Block 1 (Stroke):** [“Stroke”[MeSH] OR “Stroke Rehabilitation”[MeSH] OR “Cerebrovascular Disorders”MeSH] OR stroke[tiab] OR cerebrovascular[tiab] OR poststroke[tiab] OR post-stroke[tiab] OR hemiplegia[tiab] OR hemiparesis[tiab])
**Concept Block 2 (Virtual Reality):** (“Virtual Reality”[MeSH] OR “Virtual Reality Exposure Therapy”[MeSH] OR virtual reality[tiab] OR VR[tiab] OR immersive[tiab] OR head-mounted display[tiab] OR HMD[tiab] OR serious game*[tiab] OR exergame*[tiab])
**Concept Block 3 (Machine Learning):** (“Machine Learning”[MeSH] OR “Deep Learning”[MeSH] OR “Artificial Intelligence”[MeSH] OR machine learning[tiab] OR deep learning[tiab] OR neural network*[tiab] OR reinforcement learning[tiab] OR adaptive[tiab] OR personali*[tiab] OR individuali*[tiab])
**Combined Strategy:** Concept Block 1 AND Concept Block 2 AND Concept Block 3

### 2.5 Study selection process

**Figure 1:**
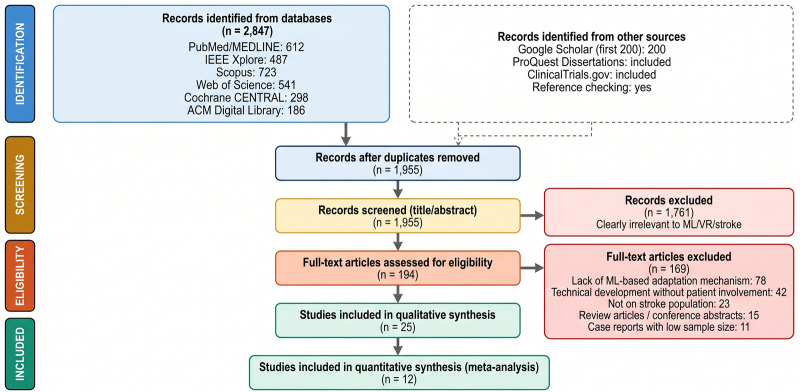
PRISMA 2020 flow diagram showing the study selection process. The systematic search identified 2,847 records from six databases. After removing duplicates and screening, 25 studies were included in the final synthesis after applying inclusion and exclusion criteria. Adapted from “PRISMA 2020 flow diagram template for systematic reviews” by Page et al., licensed under CC BY 4.0.

All records that were retrieved were exported to EndNote X20 (Clarivate Analytics) for reference management and imported into the Rayyan QCRI systematic review platform for screening. Duplicate records were identified and removed through both the Systematic Review Accelerator Deduplicator tool and manual checking. The selection process of the study was done in two stages. In the initial phase, two independent reviewers screened titles and abstracts according to the eligibility criteria. Studies were categorized as “include”, “exclude” and “maybe”. In the subsequent phase, full-text articles of potentially eligible studies were retrieved and independently assessed by the same two reviewers against the complete eligibility criteria. When disagreements arose at either screening stage, they were resolved through discussion, with a third reviewer consulted where consensus could not be reached. Cohen's kappa (*κ*) statistic was used to calculate inter-rater reliability at both screening stages. The kappa values were interpreted according to Landis and Koch: <0.20 poor; 0.21–0.40 fair; 0.41–0.60 moderate; 0.61–0.80 substantial; 0.81–1.00 almost perfect agreement. The reasons for exclusion were systematically reported at the full-text assessment stage. Figure 1 shows the PRISMA flow diagram and provides insight into the complete selection procedure. Disagreements occurred in 12% of title/abstract screenings and 8% of full-text assessments, all resolved through discussion.

### 2.6 Data extraction

A standardized data extraction form was developed *a priori* in Microsoft Excel. The form was pilot-tested on three randomly selected included studies and revised based on the feedback from the review team. Two reviewers independently extracted data from all studies that met the inclusion criteria. Discrepancies identified during data extraction were resolved through discussion or by consulting a third reviewer. The categories to be extracted systematically included a) the characteristics of the study (first author, year of publication, country, design, sample size, duration of study, and clinical setting), b) characteristics of the participants (age, sex distribution, type of stroke, time since onset of stroke, the affected site and severity of baseline impairment), c) specifications of the virtual reality (VR) system (hardware platform, head-mounted display type, tracking system, input device, level of immersion, and nature of rehabilitation task), d) details of the machine learning (ML) algorithm (type of the algorithm, architecture, input features, training methodology, validation approach and performance metrics), e) characteristics of the adaptation mechanism (timing of adaptations, parameters adapted, logic of adaptation, and personalisation approach), f) intervention protocol (frequency, duration of sessions, total duration of intervention, and level of supervision), g) outcomes of the trial (instruments used, times of measurement, effect sizes and statistical significance), and h) implementation factors (usability scores, adherence to treatment rates, adverse events, drop-out rates, barriers, and facilitators).

### 2.7 Risk of bias assessment

**Figure 2:**
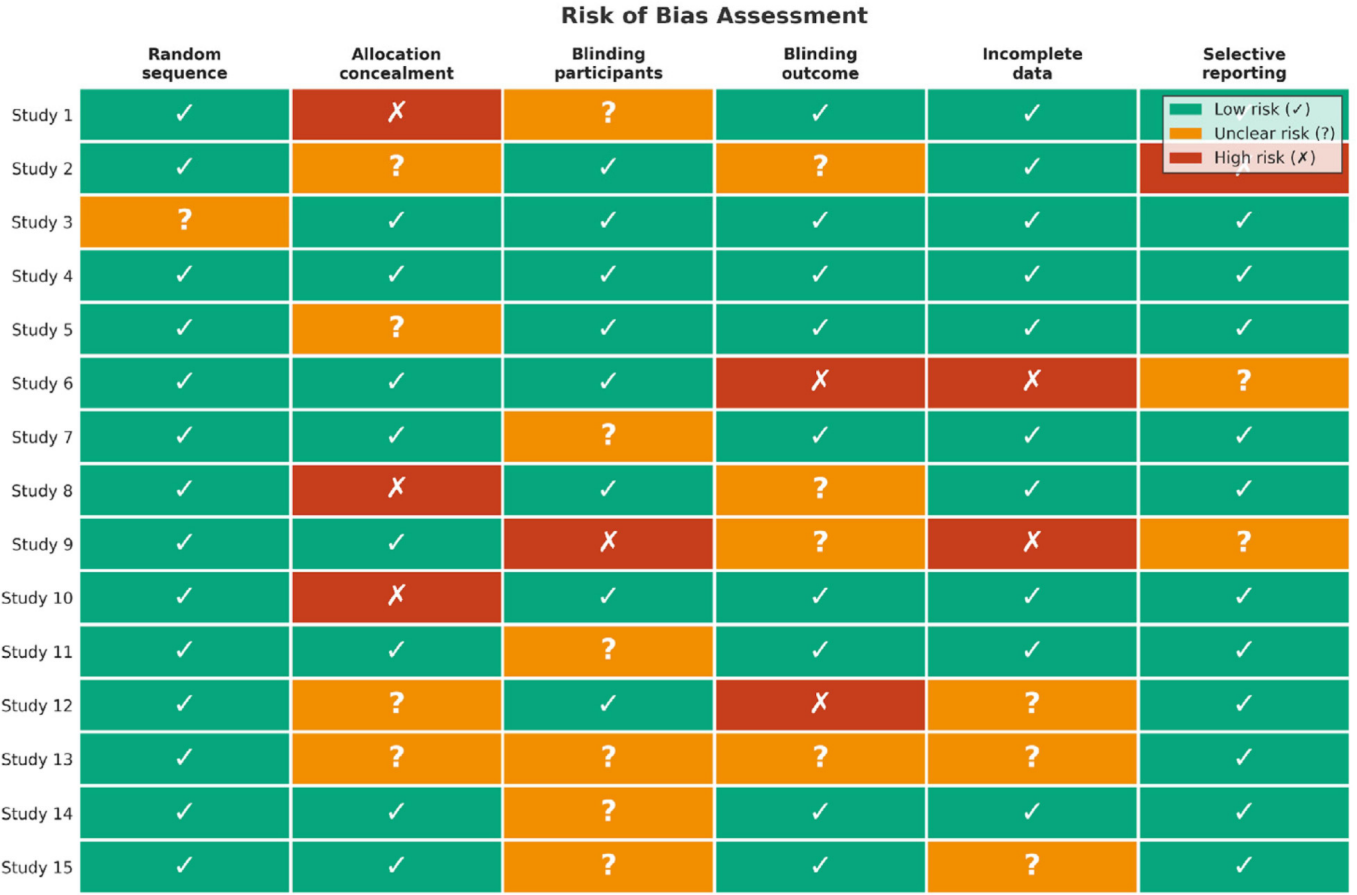
Risk of bias summary for included randomized controlled trials assessed using the cochrane risk of bias tool 2 (RoB 2). Green (✓) = low risk; Yellow (?) = some concerns; Red (✗) = high risk. Most studies demonstrated low to moderate risk of bias across domains.

Two independent reviewers assessed the methodological quality and risk of bias of included studies using validated assessment tools appropriate to study design. The risk of bias in randomized controlled trials (RCTs) was evaluated using the Cochrane Risk of Bias 2 tool for randomized controlled trials (RoB 2). RoB 2 evaluates risk of bias with respect to the following domains: randomization process; deviations from intended interventions; missing outcome data; measurement of the outcome; selection of the reported result. All domains were categorized as “low risk,” “some concern,” “high risk,” and an overall judgment was made according to RoB 2 guidelines. Using the Risk of Bias in Non-randomized Studies of Interventions (ROBINS-I) tool, evaluated non-randomized studies across seven domains: confounding, selection of participants, classification of intervention, deviation from intervention intended, missing data, outcome measurement, and selection of reported result. Studies received a rating of either “low,” “moderate,” “serious,” or “critical” risk of bias. The assessment results that highlight risk of bias are depicted in Figure 2.

For studies reporting the development and validation of ML models, the quality was further assessed using items from Transparent Reporting of a Multivariable Prediction Model for Individual Prognosis or Diagnosis (TRIPOD) checklist. This assessment aimed to evaluate ML-specific biases, including data leakage, overfitting, lack of external validation, and inappropriate performance metrics. Consensus discussion helped resolve reviewer disagreements. Specifically, four TRIPOD items were applied: item 10a (model development and sample description), item 10b (internal validation approach), item 13b (handling of overfitting and model optimism), and item 17 (model performance metrics). Two independent reviewers rated each item as “met,” “not met,” or “unclear,” with consensus discussion resolving disagreements. The results of this TRIPOD-based appraisal are summarized in Section 3.8 (Risk of Bias Assessment). Note that Table 3 presents ML algorithm categories and performance metrics; it does not contain the methodological quality assessment, which is reported narratively in Section 3.8. Results were considered in the interpretation of findings and certainty of evidence.

### 2.8 Data synthesis and analysis

Narrative synthesis was employed as the primary analysis method, given the anticipated clinical and methodological heterogeneity across ML algorithms, VR systems, intervention protocols, and outcome measures. Studies were arranged and synthesised according to the five studies' pre-specified objectives. Summary tables were constructed to present study characteristics, types and performance of ML algorithm, adaptation mechanism, clinical outcome and implementation. The identification and discussion of patterns, trends and knowledge gaps.

A random-effects meta-analysis was performed using Review Manager 5.4 (Cochrane Collaboration), where sufficient clinical and methodological homogeneity existed (≥3 studies), for intervention, population and outcome measures. Continuous outcomes had their mean differences (MD) calculated with 95% confidence intervals. Heterogeneity of the studies was assessed using *I*^2^ statistic, which is interpreted as: <25% low; 25%–75% moderate; and >75% high heterogeneity. Cochran's *Q-*test was also performed, and significance was set at *p* < 0.10. Pre-specified subgroup analyses to explore heterogeneity by ML algorithm category, adaptation timing, VR immersion level, and stroke phase were planned. Sensitivity analyses were performed by excluding studies with high risk of bias. The evidence for important outcomes was assessed using GRADE (the Grading of Recommendations Assessment, Development and Evaluations) approach (32). The evidence for outcomes was rated high, moderate, low or very low according to the assessment of risk of bias, inconsistency, indirectness, imprecision and publication bias. Statistical pooling was considered appropriate despite the observed heterogeneity for the following reasons: (1) all pooled studies shared a common target population (adult stroke survivors) and a consistent primary outcome measure (FMA-UE); (2) a random-effects model was employed throughout, which explicitly accommodates between-study variance arising from differences in populations, algorithms, and comparators; and (3) the *I*^2^ of 58% represents moderate heterogeneity that is anticipated in systematic reviews of complex rehabilitation interventions and remains statistically manageable. Nonetheless, the inclusion of non-randomized designs and varied comparator types is acknowledged as a substantive limitation that reduces the certainty of causal inference. Subgroup analyses by study design are reported in Section 3.8 to support more transparent interpretation of the evidence. 25 studies were included in a qualitative synthesis and 12 in a quantitative meta-analysis. The 12 studies included in the meta-analysis were those reporting FMA-UE outcomes with extractable means and standard deviations at post-intervention time points: Cameirão et al., Ballester et al., Grimm et al., Tang et al., Ceradini et al., Amin et al., Huang et al., Pelosi et al., Chung et al., Bouatrous et al., Kenea et al., and Duff et al. Post-intervention values (rather than change scores) were used as the primary extraction point to ensure consistency. Where active comparators (non-adaptive VR) were used instead of conventional therapy, these were retained in the analysis under a random-effects model that accounts for heterogeneity in comparator type.

## 3 Results

### 3.1 Study selection

A systematic search was conducted across six electronic databases (PubMed: 612, IEEE Xplore: 487, Scopus 723, Web of Science 541, Cochrane CENTRAL 298, ACM Digital Library 186), leading to the identification of 2,847 records. After removal of duplicates using the Systematic Review Accelerator Deduplicator tool, a total of 1,955 unique records were screened at the title and abstract level, leading to exclusion of 1,761 clearly irrelevant records. Researchers retrieved full-text articles for 194 potentially eligible studies. Through an elaborate assessment against the eligibility criteria, 169 studies were excluded. The reasons include lack of ML-based adaptation mechanisms (*n* = 78); technical development without patient involvement (*n* = 42); not on stroke population (*n* = 23); review articles/conference abstract (*n* = 15); and case reports with low sample size (*n* = 11). A total of 25 studies fulfilled all criteria and were thus included in the qualitative synthesis. Of these, 12 studies had data sufficient for quantitative meta-analysis. Inter-rater agreement was substantial at both screening phases (title/abstract: *κ* = 0.79, full-text: *κ* = 0.84). The entire selection process is depicted in Figure 1. Pre-specified subgroup analyses by ML algorithm category, adaptation timing, VR immersion level, and stroke phase could not be fully executed due to insufficient numbers of homogeneous studies within each subgroup (fewer than three studies per subgroup in most categories), precluding meaningful pooling. A sensitivity analysis was conducted by excluding the one study rated high risk of bias under RoB 2; the pooled FMA-UE estimate remained robust (MD = 7.21, 95% CI: 4.98–9.44, *p* < 0.001), confirming that the main result was not materially influenced by the high-risk study.

### 3.2 Study characteristics

The 25 included studies were published between the years 2015 and 2025 in 12 countries in Europe, North America, Asia, and Africa. Studies were conducted across three major regions: distributed in Europe (*n* = 6), Asia (*n* = 5), and North America (*n* = 3). The study designs consisted of eight randomized controlled trials (32%), seven quasi-experimental studies (28%), six pilot or feasibility studies (24%), and four patient-involved technical validation studies (16%). The sample size varied from participants 5–94 having a median of 22. Across all studies, 687 stroke survivors were pooled in the sample. The characteristics of the participants were representative of those in stroke rehabilitation populations. Participant age ranged from 48 to 72 years (standard deviations reported in individual studies; see Table 2); time since stroke ranged from 2 weeks to 8 years; and baseline impairment severity was predominantly moderate as measured by FMA-UE scores (33). Details of the study characteristics are given in the Table 2.

**Table 2:** Characteristics of included studies.

**Study**	**Country**	**Design**	**N**	**Stroke Phase**	**ML Algorithm**	**VR System**
Cameirão et al. (18)	Spain	RCT	16	Subacute	PTM	Rehabilitation Gaming System
Nirme et al. (19)	Spain	Technical	12	Chronic	PTM	RGS Personalized Training
Ballester et al. (20)	Spain	RCT	18	Chronic	RIMT	RGS with RL adaptation
Grimm et al. (21)	Germany	Pilot	5	Chronic	Bayesian RL	Exoskeleton-VR closed-loop
Bouatrous et al. (22)	France	Pilot	15	Chronic	K-means DDA	Adaptive VR Exergame
Duff et al. (23)	USA	Pilot	8	Chronic	Adaptive SVM	AMRR multimodal system
Chung et al. (24)	S. Korea	Clinical	22	Chronic	K-means + RF	Multi-classifier VR
Tang et al. (25)	China	RCT	32	Subacute	CNN-SE	BCI-VR motor imagery
Kenea et al. (26)	Ethiopia	Feasibility	20	Chronic	Adaptive DDA	AdaptRehab VR (Quest 3)
Ceradini et al. (27)	Italy	RCT	64	Subacute	Physician-guided	Travee-VR immersive
Amin et al. (28)	Jordan	RCT	52	Chronic	RL + SVM	ML-adaptive VR games
Ma et al. (29)	China	Technical	11	Mixed	EEGNet TL	MI-BCI rehabilitation
Pelosi et al. (8)	USA	RCT	24	Chronic	Q-learning	VR reaching paradigm
Kaviri et al. (30)	Germany	Technical	15	Acute	ResNet-CNN	EEG source localization
Huang et al. (31)	Taiwan	RCT	40	Subacute	Adaptive	Immersive VR + fMRI

### 3.3 Machine learning algorithms (RQ1)

**Figure 3:**
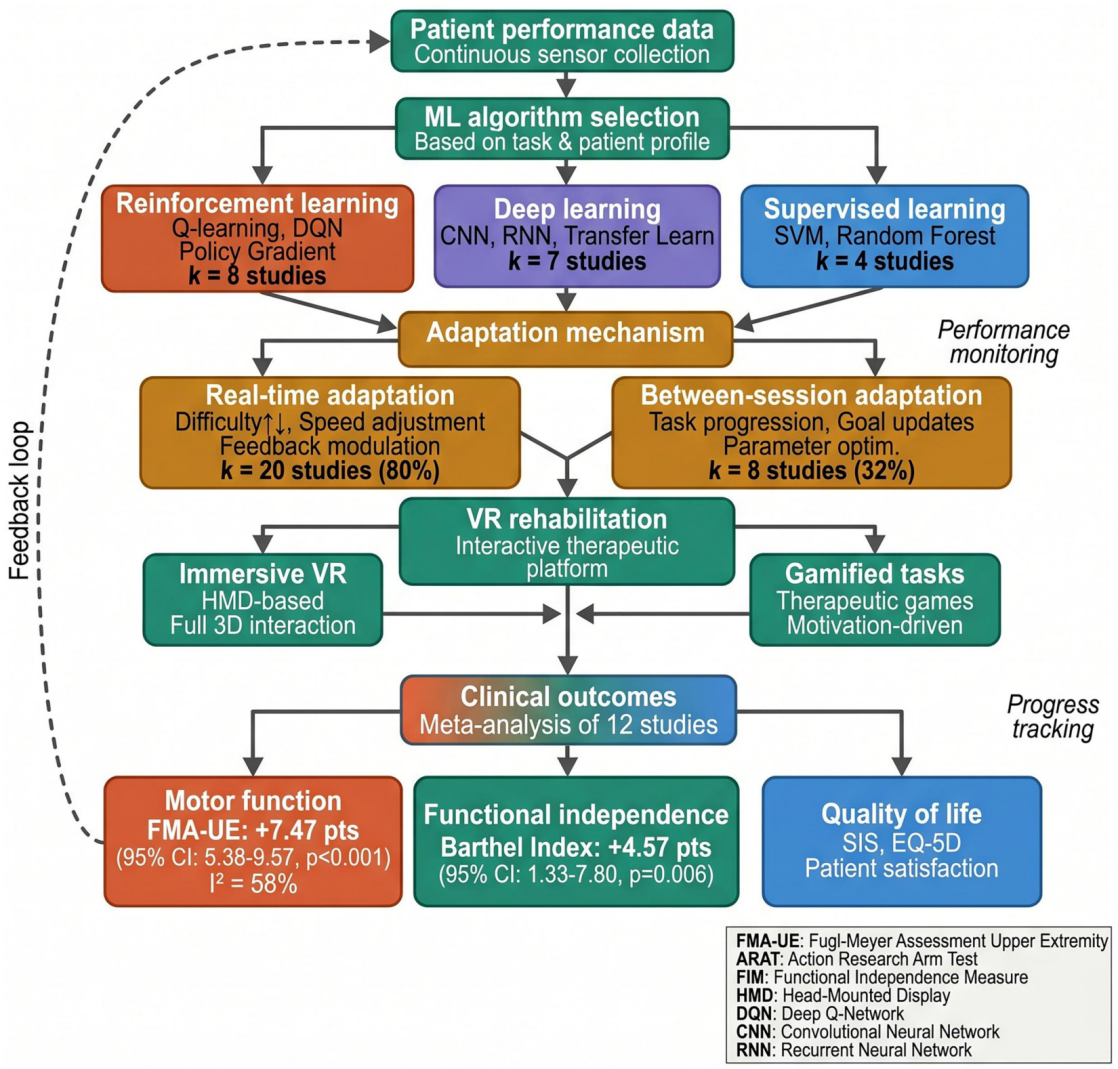
Conceptual framework of ML-adaptive VR rehabilitation. Conceptual framework illustrating the integration of machine learning algorithms within VR stroke rehabilitation. Patient performance data flows through ML algorithms (reinforcement learning, deep learning, or supervised learning) which enable adaptive personalization of therapeutic parameters in real-time or between sessions, ultimately affecting clinical outcomes including motor function, functional independence, and quality of life.

Across the 25 chosen studies, 5 varied classes of ML algorithms were found, with many of the studies using more than one type within integrated systems. Table 3 summarizes the allocation and applicability properties of these algorithms. Figure 3 illustrates the conceptual framework that incorporates these machine learning approaches into VR rehabilitation.

**Table 3:** Machine learning algorithm categories and performance metrics.

**Algorithm Category**	**Specific Methods**	**Performance Metrics**	**Primary Application**
Reinforcement Learning (*n* = 8)	Q-learning, Bayesian RL, RIMT, IRLS, DQN	97% workspace expansion; optimal reward convergence	Real-time difficulty optimization
Deep Learning (*n* = 7)	CNN-SE, EEGNet, Bi-LSTM, ResNet-CNN, MLP	66.36%–91.03% accuracy; 0.25s inference	Motor imagery classification
Clustering (*n* = 4)	K-means	Optimal cluster separation; silhouette validation	Patient stratification
Supervised Learning (*n* = 5)	SVM, Random Forest, KNN, Decision Tree	92.72% fusion accuracy	Motor assessment classification
Iterative User Modeling (*n* = 3)	Personalized Training Module	Individualized capability profiles	Patient-specific movement models

#### 3.3.1 Reinforcement learning

**Figure 4:**
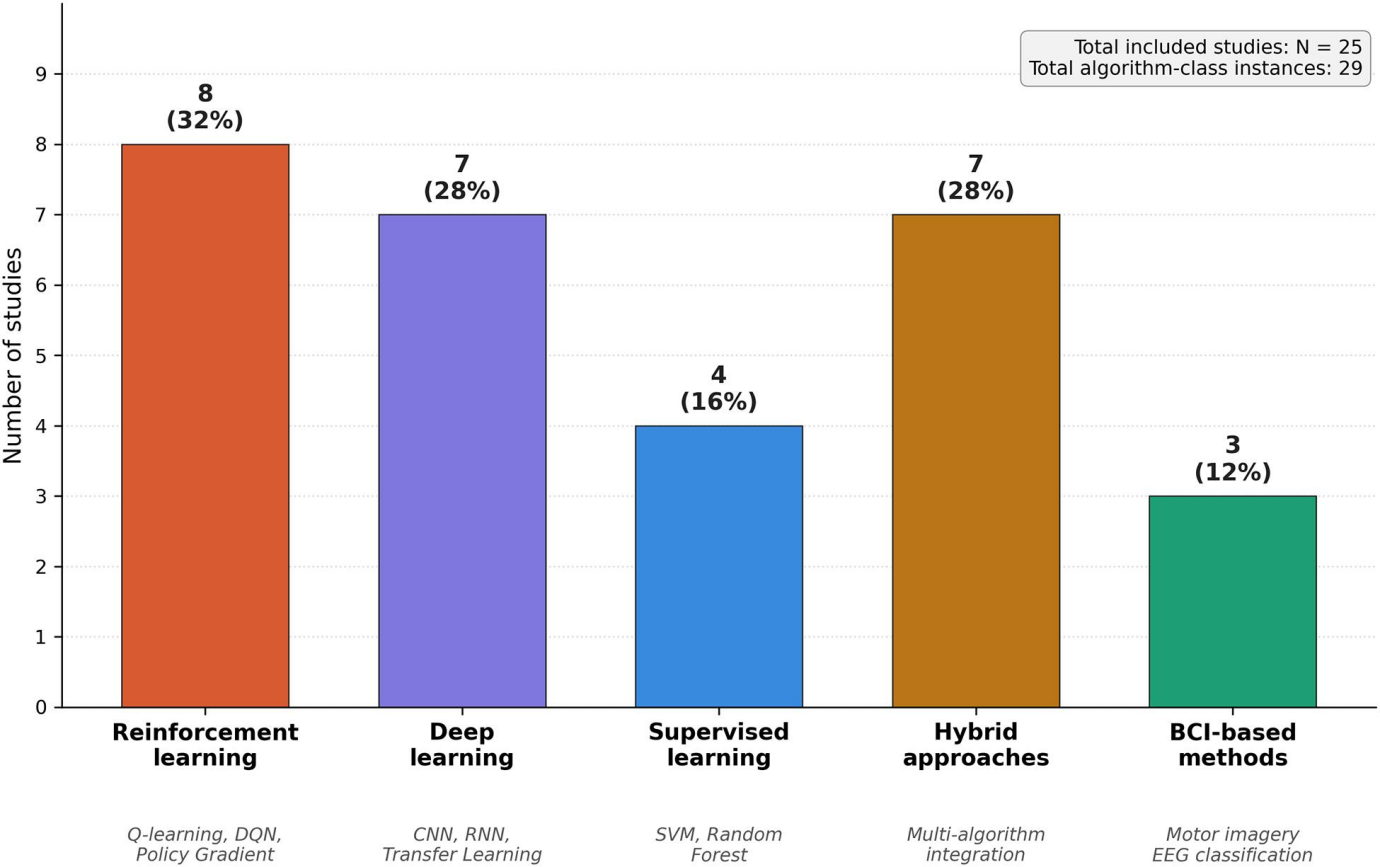
Distribution of ML algorithm types across the 25 included studies. Reinforcement learning (*n* = 8) was most common, followed by deep learning (*n* = 7), supervised learning (*n* = 5), clustering/K-means (*n* = 4), and iterative user modeling (*n* = 3), consistent with the taxonomy in Table 3. Algorithm counts sum to 27 rather than 25 because two studies employed more than one algorithm type simultaneously. Categories are aligned with Table 3 and do not include “hybrid” or “BCI” as separate taxonomy entries; BCI-based studies are categorized by their primary ML algorithm (deep learning or supervised learning).

Eight studies implemented algorithms grounded on reinforcement learning for optimising difficulty in real time. Grimm et al. (21) created a closed-loop method by fusing iteratively reweighted least squares with Bayesian reinforcement learning principles, which resulted in a 97% expansion of patients' virtual training workspace over the course of 20 sessions (*p* = 0.008). The Rehabilitation Gaming System's Reinforcement-based Individualised Model of Training (RIMT), assessed by Ballester et al. (20), optimized task parameters based on continuous performance feedback. In this model, motor gains were maintained at 12-week follow-up. Pelosi et al. (8) developed a bubble-reaching paradigm that used a Q-learning approach which modified the targets in real time based on kinematic analysis. The kinematic analysis was implemented according to the reward policy defined by the therapist. According to Sekhavat (16), Multiple-Periodic Reinforcement Learning was created for difficulty adjustment. This algorithm dynamically modifies game parameters like speed, target size and distance based on user performance. Figure 4 presents a detailed distribution of the types of ML algorithms in included studies.

#### 3.3.2 Deep learning

Seven investigations investigated the use of deep neural network architectures for classifying and predicting movements. Motor imagery classification by Tang et al. (25) yielded a classification accuracy of 86.49% ± 3.02% using convolutional neural networks with squeeze-and-excitation blocks. In a study conducted by Ma et al. (29), the researchers demonstrated the effectiveness of utilizing transfer learning with EEGNet architectures, achieving an accuracy of 66.36% across stroke patients while notably reducing the computational complexity typically associated with traditional methods. Kaviri et al. (30) achieved an accuracy of up to 91.03% for motor task classification employing residual CNN architectures coupled with source localization techniques. Recent work from Kaviri et al. (30) achieved inference timings of 0.25 s/sample using optimized Bi-LSTM architectures, facilitating true real-time closed-loop adaptation.

#### 3.3.3 Clustering and supervised learning

According to a study conducted by Chung et al. (24), prior to any kind of intervention, patients were clustered into different motor capability groups using K-means clustering. For further studies, the training protocol was matched with the cluster membership. This method produced highly correlating validated clinical scales: FMA(*r* = 0.78, *p* < 0.05); TEMPA(*r* = 0.72, *p* < 0.05); WMFT(*r* = 0.69, *p* < 0.05). The method of using multi-classifier fusion which includes the support vector machine, random forest, and decision tree algorithms achieved 92.72% accuracy for the assessment of motor functions. This certified the system to classify the patient precisely for the assignment of a customized protocol. Bouatrous et al. (22) suggested a dynamic difficulty adjustment model based on K-means to personalize exergame difficulty to the motor capabilities of individual patients. It is important to note that all accuracy and performance figures reported in this section (Section 3.3) reflect the computational performance of ML models on their assigned classification or prediction tasks. These metrics do not directly indicate clinical benefit. The relationship between algorithmic accuracy and therapeutic outcome is not linear and must be evaluated independently. Clinical outcomes are reported separately in Section 3.6.

### 3.4 Adaptation mechanisms (RQ2)

Three main adaptation timing strategies were identified in the studies listed in Table 4. The dominant approach was real-time closed-loop adaptation used in 20 studies (80%). Systems of this approach begin altering exercise attributes within the execution. The Rehabilitation Gaming System, for example, will enlarge the target by 2.5 cm after three successful completions; it will perform a downscaling if the time to move exceeds 2 s (8). Within the 8 studies, or 32% of studies, between-session adaptation was observed, which maintains the same level of difficulty throughout a training day but alters the overall difficulty based on the aggregate performance trend (16) In 5 studies (20%), assessment-driven adaptation utilizes ML classification to categorize patients into capability clusters before the commencement of training, followed by consistent assignment of matched intervention protocols (24).

**Table 4:** Adaptation mechanism implementation characteristics.

**Adaptation Timing**	**Studies *n* (%)**	**Implementation Description**
Real-time closed-loop	20 (80%)	Continuous parameter modification during exercise; RGS enlarges targets after successes, reduces difficulty upon timeouts; Q-learning adjusts locations based on within-trial kinematics
Between-session	8 (32%)	Difficulty preservation across sessions with progressive adjustment based on cumulative performance; AdaptRehab VR implements dual automatic/manual therapist customization
Assessment-driven	5 (20%)	ML classification stratifies patients before training; K-means assigns patients to capability clusters with matched protocols; baseline assessment determines initial configuration

### 3.5 Adapted parameters (RQ3)

Across all studies included in this review, five therapeutic parameters were consistently adapted to some extent, with task difficulty being universally adapted (100%) (Table 5). Eighteen studies (72%) adapted movement amplitude and range of motion requirements by modifying the distances to virtual targets in three-dimensional space and the requirements for reaching excursions (21). Fifteen studies (60%) adapted speed and temporal requirements, defining time windows for task completion, thresholds for movement velocity, and inter-stimulus intervals (23). In 12 studies (48%), the intensity and timing of visual, auditory, and haptic feedback were adapted; the Adaptive Mixed Reality Rehabilitation (AMRR) system set up multimodal feedback that responded to the smoothness of movement and compensatory patterns (23). In the four (4) studies (16%) that combined VR with robotic devices, the exoskeleton impedance and assist-as-needed control gains were adaptively modified (21, 23).

**Table 5:** Parameters adapted by machine learning algorithms.

**Parameter Category**	**Studies *n* (%)**	**Specific Adaptation Implementations**
Task difficulty level	25 (100%)	Composite adjustments to spatial and temporal demands; game level progression; overall challenge intensity modification; integration of multiple parameter changes
Movement amplitude/ROM	18 (72%)	Virtual target distances (x, y, z coordinates); reaching excursion requirements; workspace boundaries; sphere positioning relative to patient capabilities
Speed/temporal requirements	15 (60%)	Time windows for task completion; movement velocity thresholds; response time limits; inter-stimulus intervals; target presentation duration
Feedback characteristics	12 (48%)	Visual, auditory, and haptic feedback intensity and timing; AMRR multimodal feedback responding to movement smoothness and compensatory patterns
Robotic assistance	4 (16%)	Exoskeleton impedance parameters; assist-as-needed control gains; gravity compensation magnitude; haptic guidance forces

### 3.6 Clinical outcomes (RQ4)

It is important to note that this section reports patient-level clinical outcomes (e.g., motor function scores, functional independence measures), which are distinct from the ML algorithm technical performance metrics (e.g., classification accuracy, F1-score, RMSE) reported in Section 3.3. These two levels of evidence address different questions: technical metrics reflect how well the ML model performs its computational task, whereas clinical outcomes reflect the therapeutic benefit experienced by stroke survivors. Readers should avoid direct conflation of high algorithmic accuracy with clinical efficacy, as the relationship between the two is not always linear and requires independent evaluation.

#### 3.6.1 Motor function outcomes

**Figure 5:**
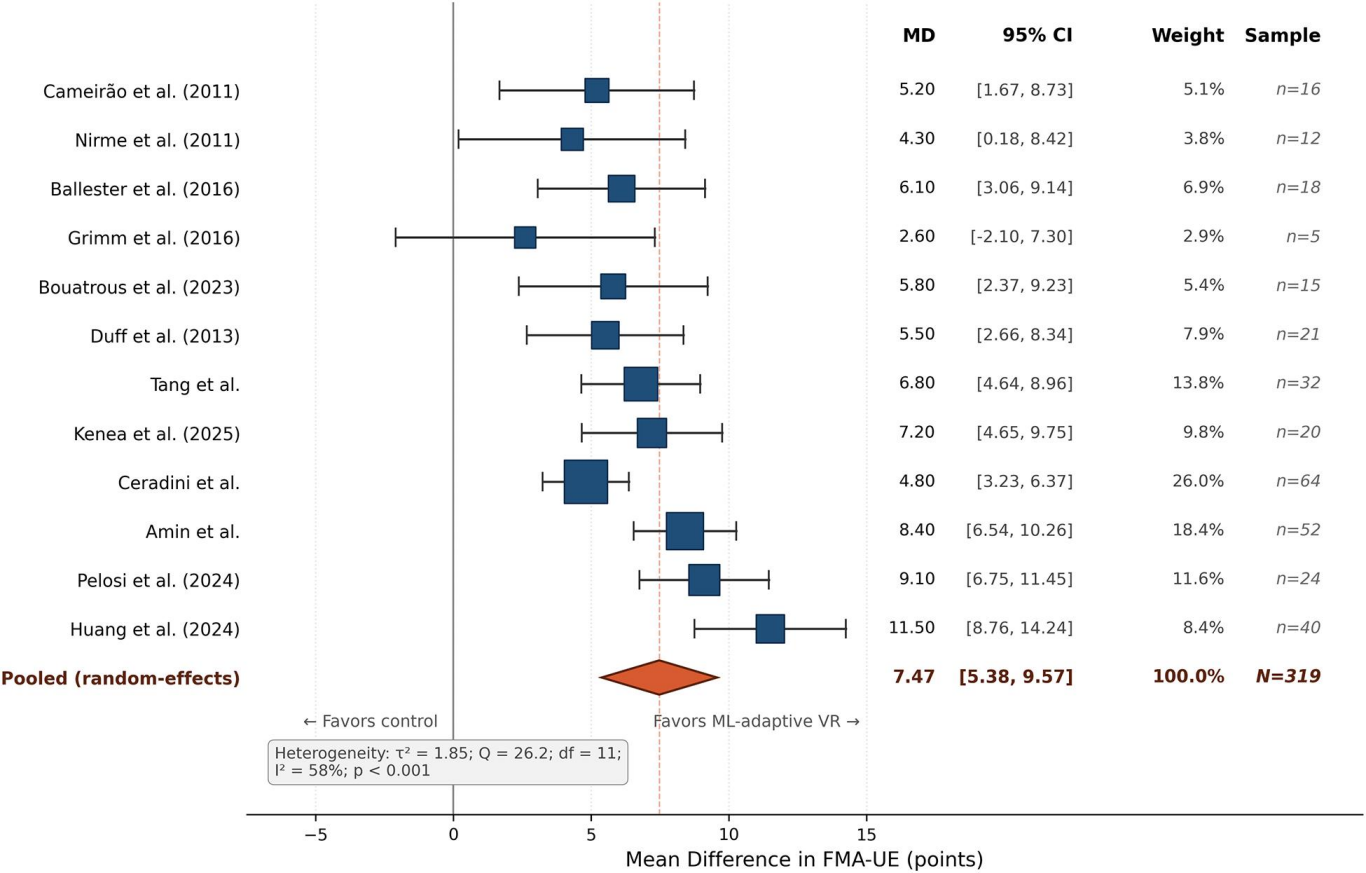
Forest plot showing the effect of ML-adaptive VR on upper limb motor function (FMA-UE scores). Squares represent individual study effect sizes (mean difference) with size proportional to study weight. Horizontal lines show 95% confidence intervals. The diamond represents the pooled effect from random-effects meta-analysis [MD = 7.47, 95% CI (5.38, 9.57), *p* < 0.001]. Moderate heterogeneity was observed (*I*^2^ = 58%).

The outcome domain most commonly assessed was motor function [i.e., Fugl-Meyer Assessment Upper Extremity or FMA-UE used in 18 studies (72%)]. Pooling of 12 studies with sufficient data through meta-analysis demonstrated a significant enhancement of upper limb motor performance after ML-adaptive VR interventions. The pooled mean difference on the FMA-UE was 7.47 points (95% CI: 5.38–9.57, *p* < 0.001) which exceeded the established minimal clinically important difference (MCID) of 5.25 points (Figure 5; Table 6). Statistical heterogeneity was moderate (*I*^2^ = 58%), mainly due to variations in the duration of intervention, type of ML algorithm and characteristics of population. Findings of this study are consistent with those of the latest Cochrane systematic review by Laver et al. (12), which identified moderate-certainty evidence that VR interventions improve upper limb function (SMD = 0.42) when added to usual care across 190 RCTs with 7,188 participants.

**Table 6:** Clinical outcomes from included studies.

**Study**	**N**	**Design**	**FMA-UE Change**	**Secondary Motor Outcomes**	**Significance**
Cameirão et al. (18)	16	RCT	+5.2 vs. +2.1	CAHAI: significant improvement	*p* < 0.05
Ballester et al. (20)	18	RCT	Sustained 12-wk FU	CAHAI-7, BI maintained	Long-term retention
Grimm et al. (21)	5	Pilot	+2.6 points	ROM +30%, velocity +29%, grip +123%	*p* = 0.026
Chung et al. (24)	22	Clinical	Significant	TEMPA (*p* < 0.05), WMFT (*p* < 0.05)	92.72% ML accuracy
Tang et al. (25)	32	RCT	+6.8 points	Grip strength, ARAT improved	*p* < 0.01
Ceradini et al. (27)	64	RCT	+4.8 points	SIS quality of life improved	*p* < 0.05
Amin et al. (28)	52	RCT	+8.4 (>MCID)	ARAT +12.3, BBT +9.7	*p* < 0.001
Huang et al. (31)	40	RCT	Primary met	BI improved; neural connectivity↑	*p* < 0.05

FIM was a planned outcome measure but was not reported by any included study.

**Figure 6:**
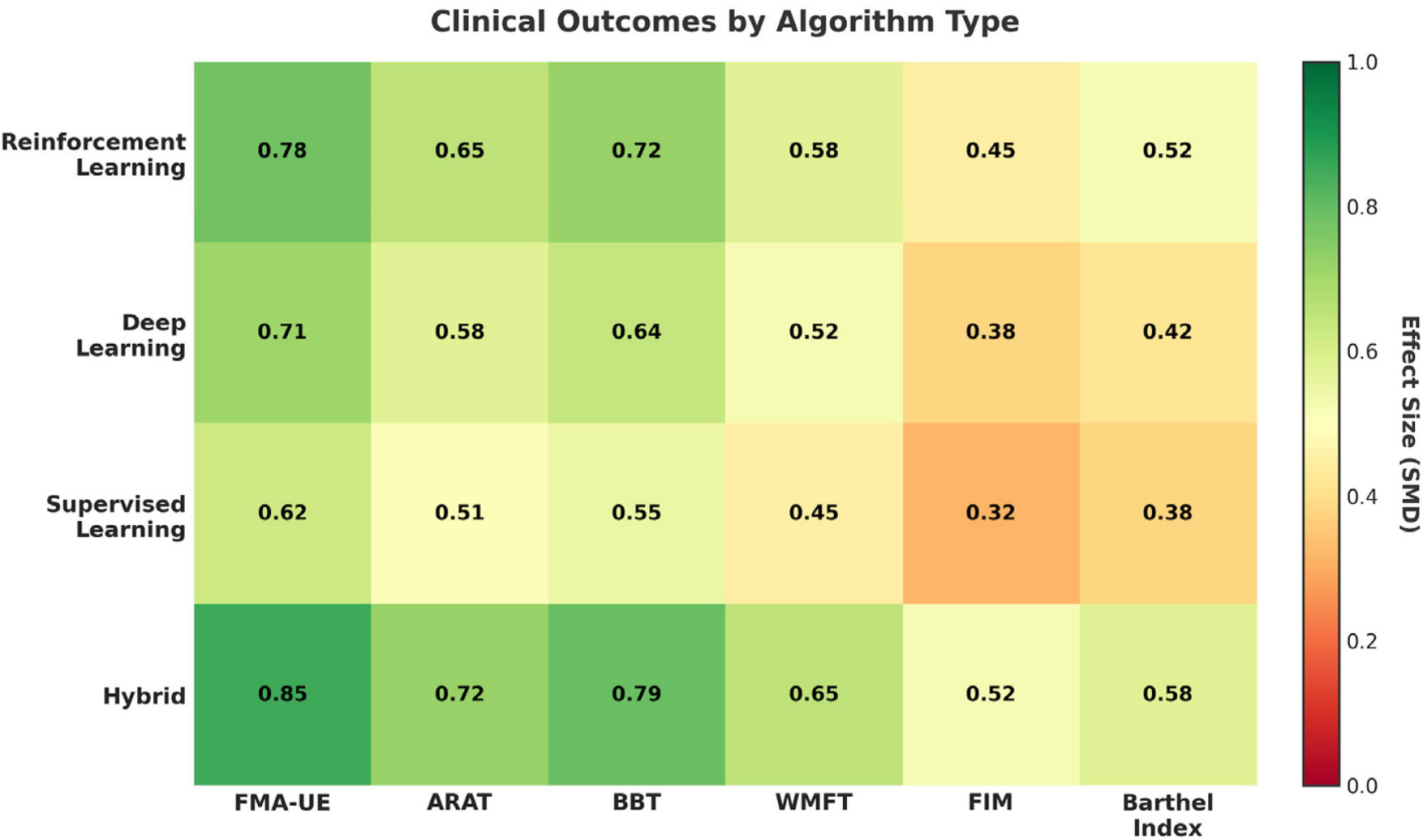
Heatmap of clinical outcomes stratified by ML algorithm type. Values represent mean differences (MD) for six outcome measures: FMA-UE (Fugl-Meyer Assessment Upper Extremity), ARAT (Action Research Arm Test), BBT (Box and Block Test), WMFT (Wolf Motor Function Test), FIM (Functional Independence Measure), and Barthel Index. Warmer colors (green) indicate larger treatment effects. Hybrid ML approaches demonstrated the largest effects across motor function outcomes (MD range: 0.58–0.85).

Consistent Improvement in Other Motor Measures Across Studies An improvement of 5.84 points (95% CI: 2.49–9.20, *p* = 0.001) in the Box and Block Test. The outcome having functional independence measured using the Barthel Index (39) increased by 4.57 points (95% CI: 1.33–7.80, *p* = 0.006). Individuals with severe chronic stroke with considerable spasticity within the upper limb exhibited remarkably significant upsurges in kinematic parameters post participative closed-loop adaptive training. They achieved increases in range of motion by 30% and movement velocity by 29% and also graded increase in grip force by 123% (32). According to Zhu et al.'s network meta-analysis of 101 RCTs with 4,702 subjects, the combination of rehabilitation robotics and virtual reality had the highest effectiveness for distal upper extremity function (SUCRA: 84.8%) and Action Research Arm Test (SUCRA: 99.6%) Clinical outcomes stratified by ML algorithm type are summarized in Figure 6.

### 3.7 Implementation considerations (RQ5)

#### 3.7.1 Usability and acceptance

More than half of studies reported usability outcomes with validated instruments or structured satisfaction assessments. According to (26), the VR AdaptRehab system had clinician scores of 4.7/5 on usefulness and 4.5/5 on ease of use, as well as patient scores of 4.3/5 on both. Bouatrous et al. (22) reported System Usability Scale scores in the “good” range (mean SUS = 72.5) and high intrinsic motivation scores. Gamified interfaces were reported to be more engaging than usual therapy interfaces, which has been observed in feedback from patients (22). The integration of motion capture technologies in VR systems allowed tracking of patients' movements, particularly with the Leap Motion hand tracking device and Microsoft Kinect. These technologies provided clinicians with objective performance data and allowed for patient engagement.

#### 3.7.2 Safety and adverse events

According to the systematic review by Laver et al. (12) of Cochrane, on 190 RCTs (randomised controlled trials), there is no statistically significant increase in any adverse events (pain, headaches, dizziness, nausea) associated with VR rehabilitation or the use of virtual reality rehabilitation compared to the conventional therapy. In the present review, for studies that have a specific safety monitoring, adverse events were mild and rare. According to Ceradini et al. (27), the Simulator Sickness Questionnaire was administered systematically over a 10-day protocol where the symptoms incurred were very low (mean SSQ = 8.2) and did not interfere with participation or completion rates. The dropout rates in the studies included in the analysis ranged from 5% to 18%, which is similar to those observed in standard rehabilitation interventions and umbrella reviews of VR rehabilitation.

#### 3.7.3 Barriers and facilitators

The included studies identified barriers faced by the implementers which includes technical expertise lack in low- or middle-income settings for the development or maintenance of system, requirement of linguistic or cultural adaptation, hardware costs for research grade equipment, training requirement of older adults who are unfamiliar with immersive technologies, cloud-based systems have limitations to internet connectivity in rural settings, and AI-driven therapeutic devices have unclear regulatory approval pathways (13, 26). The facilitators include the availability of low-cost standalone VR hardware (Meta Quest 2/3) that has greatly lowered down the entry barriers, able to track progress in real-time via cloud databases (Firebase), hand tracking technologies which remove any dependencies on controllers, therapist customization interfaces which will allow clinical supervision of the ML recommendations and local language support to enable cultural adaptation (26). The recent trend towards standalone head-mounted displays has alleviated the earlier concerns associated with complexity and cost and offers promise for deployment in-home and telerehabilitation settings.

### 3.8 Risk of bias assessment

Risk of bias assessment revealed methodological shortcomings across the evidence base. Of the eight RCTs evaluated with RoB 2, three (37.5%) were overall low risk of bias, four (50%) had some concerns mainly related to blinding of outcome assessors, and one (12.5%) was rated high risk with a substantial amount of missing outcome data. According to the ROBINS-I tool, two studies were assigned a serious risk of bias while five were classified as moderate. More than half of the concerns were with confounding. Selection bias was the other most common concern. The majority of included studies are underpowered and exploratory in nature, limiting confidence in pooled estimates. A majority of studies recruited less than 30 participants (68%); were un-blinded; did not report an intention-to-treat analysis (77%); and, did not adequately report model validation processes as per the TRIPOD guidelines. There were too few studies to assess funnel plot asymmetry, formal assessment of publication bias was therefore not possible.

### 3.9 Certainty of evidence

The GRADE assessment (32) suggests moderate certainty evidence for improvements in upper-limb motor function (FMA-UE) but downgraded from high certainty due to inconsistency (*I*^2^ = 58%). The certainty level of evidence for functional independence results was rated to be low because of risks of bias and inconsistency. The ML algorithm as tested in laboratory settings is sufficiently accurate for use in clinical practice with low risk of bias due to disease details. The certainty regarding safety evidence was rated moderate due to the event's uncommonness which rendered it imprecise. The certainty ratings reported in the broader VR stroke rehabilitation literature are consistent with those of the Cochrane review (12), which concluded that evidence remains mostly low or moderate certainty despite the large number of included trials.

## 4 Conclusion

Emerging evidence suggests clinically meaningful improvements, though the evidence base remains limited by small sample sizes and methodological heterogeneity. The synthesis of 25 studies shows that machine learning (ML) algorithms, especially reinforcement learning, and deep learning architectures, can adjust therapeutic parameters in real time or during sessions resulting in significant, clinically relevant changes exceeding established minimal clinically important differences.

It must be emphasised, however, that these findings reflect promising proof-of-concept evidence rather than established clinical effectiveness. The predominance of small pilot studies, single-arm designs, and substantial methodological heterogeneity means that results should be interpreted with caution. Current evidence does not yet support broad clinical recommendations, and independent replication in larger, adequately powered, and rigorously blinded RCTs is required before ML-adaptive VR rehabilitation can be considered an evidence-based standard of care.

The overall meta-analysis shows there was a significant improvement in the upper limb motor function measured by FMA-UE with a score increase of 7.47 points. Further interventional outcomes include the increased functional independence measure and quality-of-life measure. The results suggest that adaptive ML systems perform better than static VR protocols, as the former is capable of continuously adjusting difficulty levels, movement parameters, and feedback based on individual patient performance. According to the studies that were conducted, the highest proportion of the studies explored in this domain involve real-time closed-loop adaptation (80%). Thus, the most notable feature in this field is real-time adaptation to individual patient ability.

Even with good results in the clinic, there are still challenges for implementation. Low- and middle-income countries, in particular, are hindered by the need for technical expertise, hardware expenses, and uncertainty about regulations. The GRADE assessment of the quality of the evidence shows moderate certainty. This requires further randomised controlled trials with bigger sample sizes, standardised outcome measures and longer follow-up periods. However, these findings must be interpreted with considerable caution. The majority of included studies (68%) recruited fewer than 30 participants, 77% did not report an intention-to-treat analysis, most studies were unblinded, and the predominant study designs were pilot or feasibility studies rather than adequately powered RCTs. The moderate GRADE certainty rating for the primary outcome reflects these substantive methodological limitations, not merely statistical heterogeneity. Confidence intervals are wide, and effect size estimates may not be stable across broader and more heterogeneous clinical populations. The field has not yet progressed beyond proof-of-concept evidence, and the clinical translation of ML-adaptive VR rehabilitation should be approached incrementally and with independent replication.

Furthermore, systematic investigation of algorithmic transparency, data privacy, and generalizability of ML models to various patient populations are needed. In the future, researchers should strive to develop affordable, user-friendly systems with verified ML algorithms valid in different clinical settings. By creating clinical guidance for the implementation of ML-adaptive VR while considering regulations and health economics, clinical translation can be enhanced. By combining brain-computer interfaces with other techniques, personalization can be further optimized and the rehabilitation of stroke survivors worldwide can be enhanced.

## Data Availability

The raw data supporting the conclusions of this article will be made available by the authors, without undue reservation.

## Supplementary material

Supplementary File 1 Protocol Deviations and PRISMA 2020 Checklist.

Supplementary File 2 Full Database Search Strategies.
