# Supplementary material for: Machine learning-based adaptive personalization in virtual reality stroke rehabilitation: a systematic review
Source: Front Rehabil Sci. 2026 Jun 16;7:1827658. doi: 10.3389/fresc.2026.1827658 (PMC13314780; doi:10.3389/fresc.2026.1827658)
Supplement: Supplementary File 1 — Protocol Deviations and PRISMA 2020 Checklist. [file Supplementaryfile1.docx]

**Supplementary Appendix S1**

**Protocol Deviations & PRISMA 2020 Checklist**

*Machine learning-based adaptive personalization in virtual reality stroke rehabilitation: a systematic review*

Al Tawil A, Mohd Hashim SH, Aburub A, Darabseh MZ, Prémusz V, Hock M

Frontiers in Rehabilitation Sciences, 2026 — DOI: 10.3389/fresc.2026.1827658

# Part A — Protocol Deviations

This systematic review was prospectively registered on PROSPERO (International Prospective Register of Systematic Reviews; identifier CRD420261298450). The protocol is publicly accessible at https://www.crd.york.ac.uk/PROSPERO/view/CRD420261298450. In accordance with the Cochrane Handbook and PRISMA 2020 recommendations, all deviations between the registered protocol and the conduct or reporting of the final review are transparently documented below, with justifications.

| **Element** | **As registered (PROSPERO)** | **As reported in final manuscript** | **Justification / explanation** |
| --- | --- | --- | --- |
| **Review title** | Machine Learning-Based Adaptive Personalization in Virtual Reality Stroke Rehabilitation: A Systematic Review of Effectiveness, Feasibility, and Implementation | Machine learning-based adaptive personalization in virtual reality stroke rehabilitation: a systematic review | Title shortened during manuscript preparation to conform to Frontiers' title-length style guidelines. The substantive scope (effectiveness, feasibility, and implementation) is preserved across the manuscript's objectives, methods, and research questions (RQ1–RQ5). |
| **Author team** | Three authors registered on PROSPERO: Mohammad Z. Darabseh, Aseel Aburub, Arar Al Tawil | Six authors on final manuscript: Arar Al Tawil, Siti Hazyanti Mohd Hashim, Aseel Aburub, Mohammad Z. Darabseh, Viktória Prémusz, Márta Hock | Three additional co-authors (SHM, VP, MH) were added during the protocol-execution phase to contribute methodological supervision (SHM) and additional expertise in rehabilitation sciences and meta-analysis interpretation (VP, MH). All added authors meet ICMJE authorship criteria and contributed to manuscript drafting/review. |
| **Search end date** | Initially planned through Q3 2025 | Search extended through 15 December 2025 | Search window was extended by approximately three months to capture the most recent eligible literature published before manuscript submission, given the rapid pace of publication in this field. |
| **Risk-of-bias tools** | Cochrane Risk of Bias 2 (RoB 2) for randomized controlled trials and ROBINS-I for non-randomized studies | Cochrane RoB 2 + ROBINS-I as planned, with the addition of TRIPOD checklist items 1, 6b, 10b, and 13b for ML-prediction-model components of included studies | TRIPOD-Cluster guidance was incorporated post-hoc to address the prediction-model nature of several included studies that report model-development and validation metrics. The addition was made before any judgments were rendered and applied uniformly across studies. |
| **Subgroup analyses** | Pre-specified subgroups: stroke phase (acute/subacute/chronic), algorithm class (RL/DL/SL), and immersion level | Subgroups reported as planned. Additional sensitivity analyses (leave-one-out, removal of high-risk studies) were performed | Sensitivity analyses were added during the synthesis phase to test robustness of the pooled estimate. These additions are reported transparently in Section 3 and the certainty-of-evidence assessment. |
| **Reporting structure** | Standard IMRaD with separate Discussion and Conclusion sections | Combined Conclusion section integrating interpretation and conclusions; no separate Discussion section | During manuscript preparation, the Discussion content was integrated into Section 4 (Conclusion) to streamline interpretation of results. All discussion elements (limitations, comparison with prior reviews, implementation considerations, and future research directions) are present and clearly demarcated within Section 4. |

*All deviations were minor in nature, made transparently before any judgments were rendered (where they affected methodology), and are reported here to support reproducibility and adherence to PRISMA 2020 item 24c.*

# Part B — PRISMA 2020 Checklist

The 27-item PRISMA 2020 checklist is reported in full below, indicating the location in the manuscript where each item is addressed. Section numbers refer to the main text; Tables and Figures are numbered as in the published article. Page numbers will be updated to the final published version after typesetting.

| **Section / Topic** | **Item #** | **Checklist item** | **Location in manuscript** |
| --- | --- | --- | --- |
| **TITLE** |  |  |  |
| Title | 1 | Identify the report as a systematic review. | Title page; subtitle 'a systematic review' |
| **ABSTRACT** |  |  |  |
| Abstract | 2 | See the PRISMA 2020 for Abstracts checklist. | Abstract – structured under Background, Objective, Methods, Results, Conclusion |
| **INTRODUCTION** |  |  |  |
| Rationale | 3 | Describe the rationale for the review in the context of existing knowledge. | Section 1.1 Background and rationale |
| Objectives | 4 | Provide an explicit statement of the objective(s) or question(s). | Section 1.3 Objectives; Research Questions RQ1–RQ5 |
| **METHODS** |  |  |  |
| Eligibility criteria | 5 | Specify inclusion and exclusion criteria. | Section 2.2 Eligibility criteria (PICO framework table) |
| Information sources | 6 | Specify all databases, registers, and other sources searched. | Section 2.3 Databases used |
| Search strategy | 7 | Present the full search strategies for all sources. | Section 2.4 Search strategy; full strings in Supplementary Appendix S2 |
| Selection process | 8 | Specify the methods used to decide inclusion of studies. | Section 2.5 Study selection process |
| Data collection process | 9 | Specify methods used to collect data from reports. | Section 2.6 Data extraction |
| Data items | 10a | List and define all outcomes for which data were sought. | Section 2.6; Table 2 |
| Data items | 10b | List and define all other variables for which data were sought. | Section 2.6 (a–h categories) |
| Study risk of bias | 11 | Specify methods used to assess risk of bias. | Section 2.7 Risk of bias assessment (RoB 2, ROBINS-I) |
| Effect measures | 12 | Specify effect measures used for each outcome. | Section 2.8; Mean differences with 95% CI |
| Synthesis methods | 13a | Describe processes to decide which studies were eligible for each synthesis. | Section 2.8 Synthesis methods |
| Synthesis methods | 13b | Describe methods to prepare data for synthesis. | Section 2.8 |
| Synthesis methods | 13c | Describe methods to tabulate or visually display results. | Section 2.8; Tables 1–6; Figures 1–6 |
| Synthesis methods | 13d | Describe methods of statistical synthesis (random-effects, I²). | Section 2.8; random-effects model |
| Synthesis methods | 13e | Describe methods to explore heterogeneity. | Section 2.8; I² statistic, subgroup analyses |
| Synthesis methods | 13f | Describe sensitivity analyses. | Section 3.6; leave-one-out reported |
| Reporting bias | 14 | Describe methods used to assess risk of bias due to missing results. | Section 2.7 / 3.8 (publication bias addressed; funnel plots infeasible due to study count) |
| Certainty assessment | 15 | Describe methods used to assess certainty in the body of evidence. | Section 2.9 / 3.9 GRADE approach |
| **RESULTS** |  |  |  |
| Study selection | 16a | Describe the results of the search and selection process. | Section 3.1; Figure 1 PRISMA flow diagram |
| Study selection | 16b | Cite studies that might appear to meet inclusion criteria but were excluded. | Section 3.1; reasons documented at full-text stage |
| Study characteristics | 17 | Cite each included study and present its characteristics. | Section 3.2; Tables 1, 2, 3 |
| Risk of bias in studies | 18 | Present assessments of risk of bias for each included study. | Section 3.8; Figure 2 RoB heatmap |
| Results of individual studies | 19 | For all outcomes, present summary statistics for each study. | Section 3.6; Table 5/6; Figure 3 forest plot |
| Results of syntheses | 20a | Briefly summarize the characteristics and risk of bias among studies contributing to each synthesis. | Section 3.6.1; Section 3.8 |
| Results of syntheses | 20b | Present results of all statistical syntheses conducted (effect estimates, CIs, heterogeneity). | Section 3.6.1; FMA-UE MD = 7.47 (95% CI 5.38–9.57), I² = 58% |
| Results of syntheses | 20c | Present results of all investigations of possible causes of heterogeneity. | Section 3.6; subgroup analyses by algorithm class |
| Results of syntheses | 20d | Present results of all sensitivity analyses. | Section 3.6; leave-one-out sensitivity |
| Reporting biases | 21 | Present assessments of risk of bias due to missing results. | Section 3.8 |
| Certainty of evidence | 22 | Present assessments of certainty in the body of evidence for each outcome. | Section 3.9 GRADE certainty summary |
| **DISCUSSION** |  |  |  |
| Discussion | 23a | Provide a general interpretation of the results in the context of other evidence. | Section 4 Conclusion (integrates discussion of findings vs. prior reviews) |
| Discussion | 23b | Discuss any limitations of the evidence. | Section 4 (limitations paragraph) |
| Discussion | 23c | Discuss any limitations of the review processes used. | Section 4 (review-process limitations) |
| Discussion | 23d | Discuss implications of the results for practice, policy, and future research. | Section 4 (implementation and future research) |
| **OTHER INFORMATION** |  |  |  |
| Registration and protocol | 24a | Provide registration information for the review. | Abstract; PROSPERO CRD420261298450 |
| Registration and protocol | 24b | Indicate where the review protocol can be accessed. | https://www.crd.york.ac.uk/PROSPERO/view/CRD420261298450 |
| Registration and protocol | 24c | Describe and explain any amendments to information provided at registration. | This Supplementary Appendix S1 documents all deviations |
| Support | 25 | Describe sources of financial or non-financial support for the review. | Funding section – no external funding received |
| Competing interests | 26 | Declare any competing interests. | Conflict of Interest section – none declared |
| Availability of data, code, materials | 27 | Report which of the following are publicly available and where: data; analytic code; other materials. | Data Availability statement; raw data available from authors on reasonable request |

*Reference: Page MJ, McKenzie JE, Bossuyt PM, Boutron I, Hoffmann TC, Mulrow CD, et al. The PRISMA 2020 statement: an updated guideline for reporting systematic reviews. BMJ 2021;372:n71. doi:10.1136/bmj.n71*
