# Supplementary material for: Machine learning-based adaptive personalization in virtual reality stroke rehabilitation: a systematic review
Source: Front Rehabil Sci. 2026 Jun 16;7:1827658. doi: 10.3389/fresc.2026.1827658 (PMC13314780; doi:10.3389/fresc.2026.1827658)
Supplement: Supplementary File 2 — Full Database Search Strategies. [file Supplementaryfile2.docx]

**Supplementary Appendix S2**

**Full Search Strategies**

*Machine learning-based adaptive personalization in virtual reality stroke rehabilitation: a systematic review*

Al Tawil A, Mohd Hashim SH, Aburub A, Darabseh MZ, Premusz V, Hock M

Frontiers in Rehabilitation Sciences, 2026 - DOI: 10.3389/fresc.2026.1827658

**Overview**

This appendix documents the complete search strategies used to identify studies for the systematic review on machine learning-based adaptive personalization in virtual reality (VR) stroke rehabilitation. The strategies were developed in consultation with an information specialist and adapted to the search syntax of each database.

The search combined three conceptual blocks using Boolean AND: (a) stroke and other cerebrovascular incidents, (b) virtual reality technology and applications, and (c) machine learning / artificial intelligence. Within each block, terms were joined using Boolean OR, combining MeSH (or platform-equivalent controlled vocabulary) with free-text keywords and truncation where supported.

Searches were executed for records published between 1 January 2015 and 15 December 2025. Each database search was exported in full to EndNote X20 for reference management and subsequently imported into Rayyan QCRI for de-duplication and screening.

**1. PubMed / MEDLINE**

**Platform: U.S. National Library of Medicine**

Search date and range: Searched 15 December 2025; date range 01 January 2015 - 15 December 2025

Notes: MeSH terms combined with free-text keywords using Boolean operators. Filters: English language; Humans.

**Search syntax:**

("Stroke"[Mesh] OR "Cerebrovascular Disorders"[Mesh] OR "Brain Ischemia"[Mesh] OR stroke[tiab] OR cerebrovascular[tiab] OR "cerebral infarction"[tiab] OR poststroke[tiab] OR "post-stroke"[tiab])

AND

("Virtual Reality"[Mesh] OR "Virtual Reality Exposure Therapy"[Mesh] OR "virtual reality"[tiab] OR "VR"[tiab] OR "immersive virtual"[tiab] OR "head-mounted display"[tiab] OR HMD[tiab] OR "serious game"[tiab] OR exergam*[tiab])

AND

("Machine Learning"[Mesh] OR "Deep Learning"[Mesh] OR "Artificial Intelligence"[Mesh] OR "Neural Networks, Computer"[Mesh] OR "machine learning"[tiab] OR "deep learning"[tiab] OR "reinforcement learning"[tiab] OR "supervised learning"[tiab] OR "artificial intelligence"[tiab] OR "neural network*"[tiab] OR "adaptive algorithm*"[tiab] OR "personali*ed"[tiab])

*Records retrieved: 612*

**2. IEEE Xplore**

**Platform: IEEE Digital Library**

Search date and range: Searched 15 December 2025; date range 2015 - 2025

Notes: Command-search syntax. Filters: Conferences, Journals, Early Access; English language.

**Search syntax:**

("All Metadata":stroke OR "All Metadata":cerebrovascular OR "All Metadata":poststroke OR "All Metadata":"post-stroke")

AND

("All Metadata":"virtual reality" OR "All Metadata":VR OR "All Metadata":"head-mounted display" OR "All Metadata":HMD OR "All Metadata":"immersive virtual")

AND

("All Metadata":"machine learning" OR "All Metadata":"deep learning" OR "All Metadata":"reinforcement learning" OR "All Metadata":"artificial intelligence" OR "All Metadata":"neural network" OR "All Metadata":"adaptive")

*Records retrieved: 487*

**3. Scopus**

**Platform: Elsevier**

Search date and range: Searched 15 December 2025; date range PUBYEAR > 2014 AND PUBYEAR < 2026

Notes: TITLE-ABS-KEY search with truncation. Filters: DOCTYPE(ar) OR DOCTYPE(cp); LANGUAGE(English).

**Search syntax:**

TITLE-ABS-KEY ( ( stroke OR cerebrovascular OR poststroke OR "post-stroke" OR "cerebral infarction" OR "brain ischemia" )

AND ( "virtual reality" OR "VR" OR "immersive virtual" OR "head-mounted display" OR HMD OR "serious game*" OR exergam* )

AND ( "machine learning" OR "deep learning" OR "reinforcement learning" OR "supervised learning" OR "artificial intelligence" OR "neural network*" OR "adaptive algorithm*" OR personali?ed ) )

*Records retrieved: 723*

**4. Web of Science Core Collection**

**Platform: Clarivate Analytics**

Search date and range: Searched 15 December 2025; date range 2015 - 2025

Notes: Topic (TS) search across title, abstract, keywords, and KeyWords Plus.

**Search syntax:**

TS = ( stroke OR cerebrovascular OR poststroke OR "post-stroke" OR "brain ischemia" )

AND TS = ( "virtual reality" OR VR OR "immersive virtual" OR "head-mounted display" OR HMD OR "serious game*" OR exergam* )

AND TS = ( "machine learning" OR "deep learning" OR "reinforcement learning" OR "supervised learning" OR "artificial intelligence" OR "neural network*" OR "adaptive algorithm*" OR personali?ed )

*Records retrieved: 541*

**5. Cochrane Central Register of Controlled Trials (CENTRAL)**

**Platform: Cochrane Library / Wiley**

Search date and range: Searched 15 December 2025; date range 2015 - 2025

Notes: Cochrane search syntax. Filter: Trials.

**Search syntax:**

#1 MeSH descriptor: [Stroke] explode all trees

#2 (stroke or cerebrovascular or poststroke or "post-stroke"):ti,ab,kw

#3 #1 OR #2

#4 MeSH descriptor: [Virtual Reality] explode all trees

#5 ("virtual reality" or VR or "immersive virtual" or "head-mounted display" or HMD):ti,ab,kw

#6 #4 OR #5

#7 MeSH descriptor: [Machine Learning] explode all trees

#8 ("machine learning" or "deep learning" or "reinforcement learning" or "artificial intelligence" or "neural network*" or adaptive):ti,ab,kw

#9 #7 OR #8

#10 #3 AND #6 AND #9

*Records retrieved: 298*

**6. ACM Digital Library**

**Platform: Association for Computing Machinery**

Search date and range: Searched 15 December 2025; date range 2015 - 2025

Notes: Advanced search in [Abstract] OR [Title] OR [Author keywords].

**Search syntax:**

Abstract:( stroke OR cerebrovascular OR poststroke OR "post-stroke" )

AND Abstract:( "virtual reality" OR VR OR "immersive virtual" OR "head-mounted display" OR HMD OR exergame* )

AND Abstract:( "machine learning" OR "deep learning" OR "reinforcement learning" OR "artificial intelligence" OR "neural network*" OR adaptive )

*Records retrieved: 186*

**Grey-Literature Sources**

**1. Google Scholar**

First 200 results screened. Search string: "stroke" AND "virtual reality" AND ("machine learning" OR "deep learning" OR "reinforcement learning"). Date range 2015 - 2025.

**2. ProQuest Dissertations and Theses Global**

Subject area: Medicine, Computer Science. Search string: ab(stroke) AND ab("virtual reality") AND ab("machine learning" OR "deep learning" OR "reinforcement learning"). Date range 2015 - 2025.

**3. ClinicalTrials.gov**

Condition: Stroke. Other terms: "virtual reality" AND ("machine learning" OR "adaptive"). Status: All studies. Date range 01/2015 - 12/2025.

**Citation Handling and De-duplication**

All retrieved records were exported in RIS or BibTeX format and imported into EndNote X20 (Clarivate Analytics) for centralized reference management. Records were then transferred to Rayyan QCRI for blinded title/abstract and full-text screening by two independent reviewers. Duplicate records were identified and removed using a two-step process: (i) automated detection with the Systematic Review Accelerator Deduplicator (Bond University), and (ii) manual verification of borderline duplicates.

Reference lists of all included studies and any relevant systematic reviews were manually screened (backward citation chasing) to capture additional records not retrieved through database searches. Where studies reported insufficient methodological or outcome data, corresponding authors were contacted by email.

**Summary of Records Retrieved**

The complete count of records retrieved from each of the six electronic databases is shown below. These counts correspond to the figures reported in Section 3.1 of the main manuscript and form the basis of the PRISMA 2020 flow diagram (Figure 1).

| **Database** | **Records retrieved** | **Date searched** |
| --- | --- | --- |
| PubMed / MEDLINE | 612 | 15 December 2025 |
| IEEE Xplore | 487 | 15 December 2025 |
| Scopus | 723 | 15 December 2025 |
| Web of Science Core Collection | 541 | 15 December 2025 |
| Cochrane CENTRAL | 298 | 15 December 2025 |
| ACM Digital Library | 186 | 15 December 2025 |
| **TOTAL (six databases)** | **2,847** | **-** |

After de-duplication using the Systematic Review Accelerator Deduplicator and manual verification, 1,955 unique records remained for title/abstract screening. 1,761 records were excluded at this stage. Full-text articles were retrieved for 194 potentially eligible studies, of which 169 were excluded with the following documented reasons: lack of ML-based adaptation mechanisms (n = 78); technical development without patient involvement (n = 42); not on stroke population (n = 23); review articles or conference abstracts (n = 15); and case reports with very small sample sizes (n = 11). A total of 25 studies fulfilled all eligibility criteria and were included in the qualitative synthesis; 12 of these contained data sufficient for inclusion in the quantitative meta-analysis.
